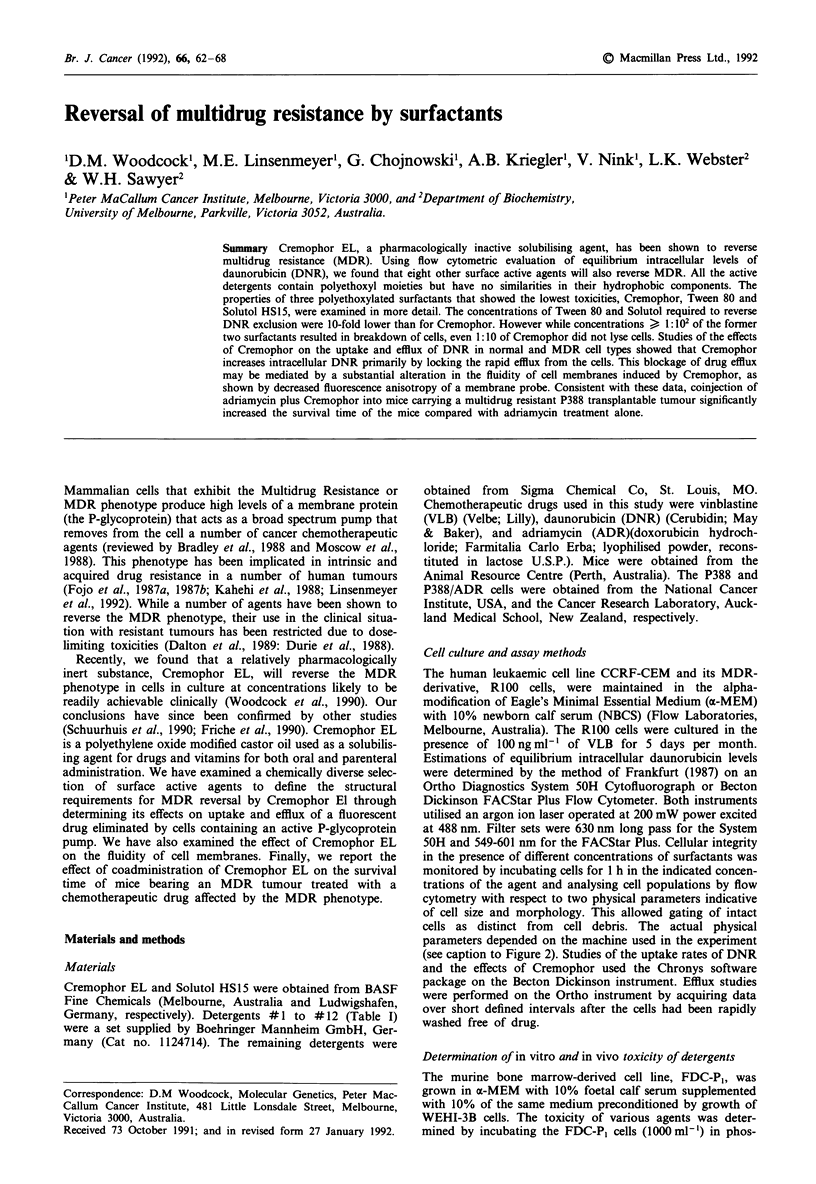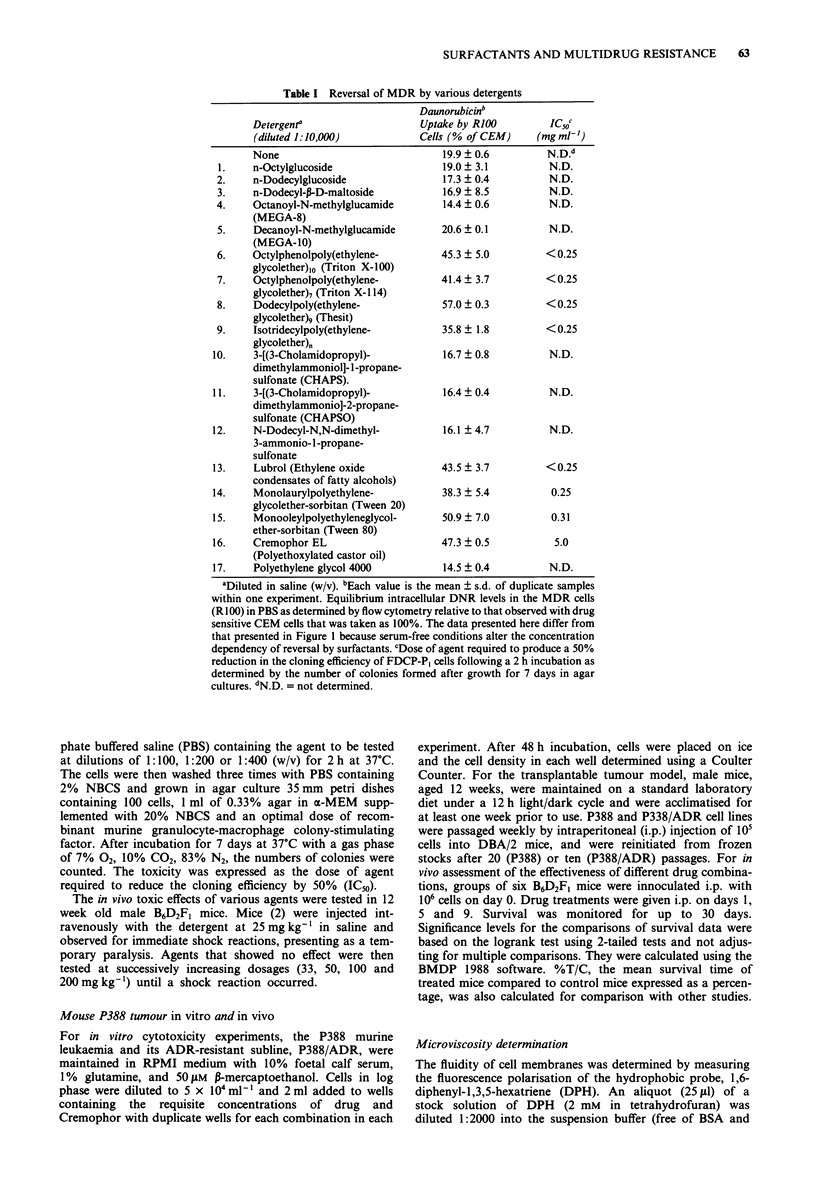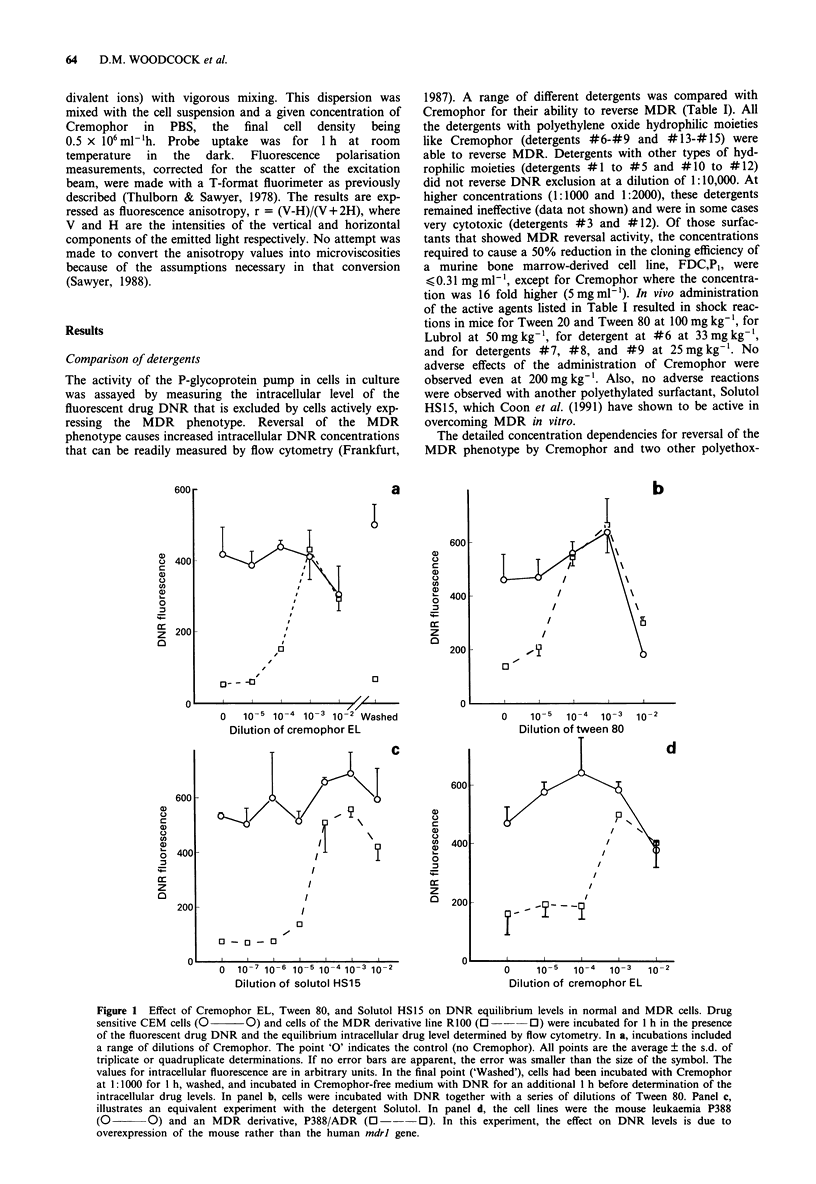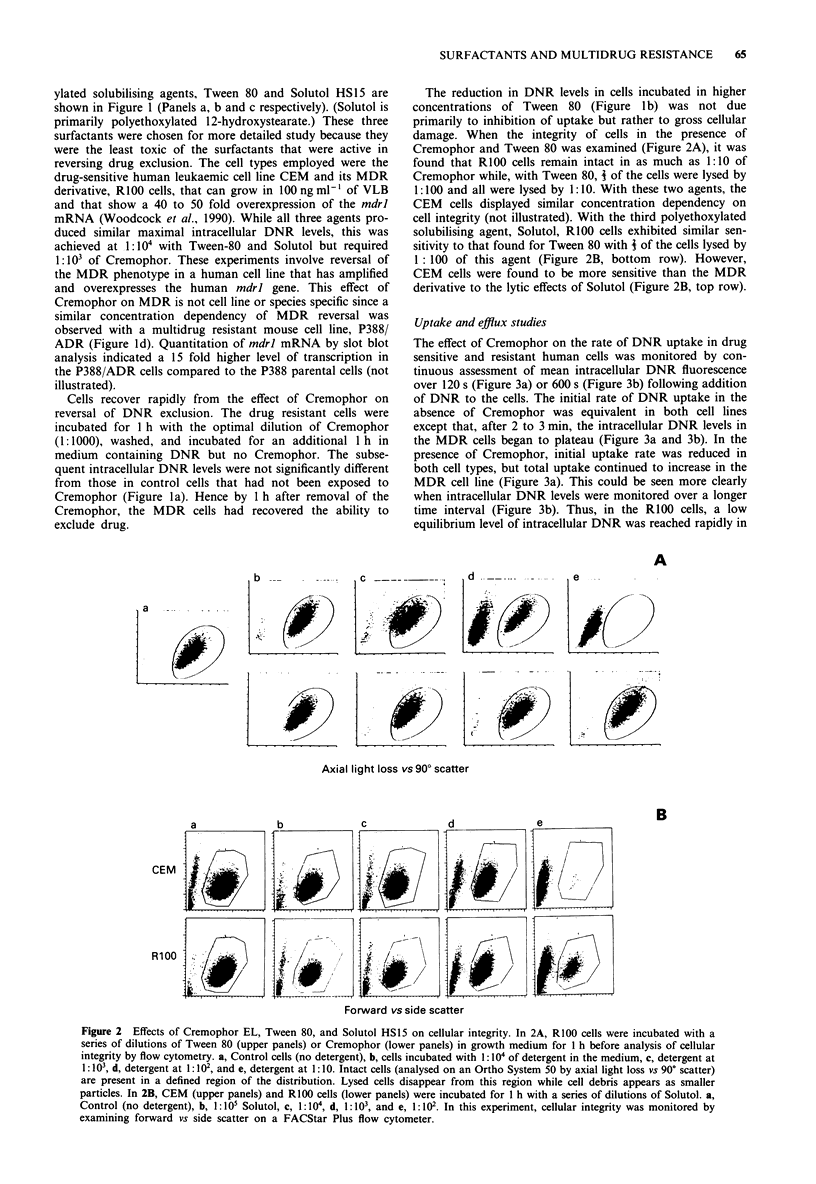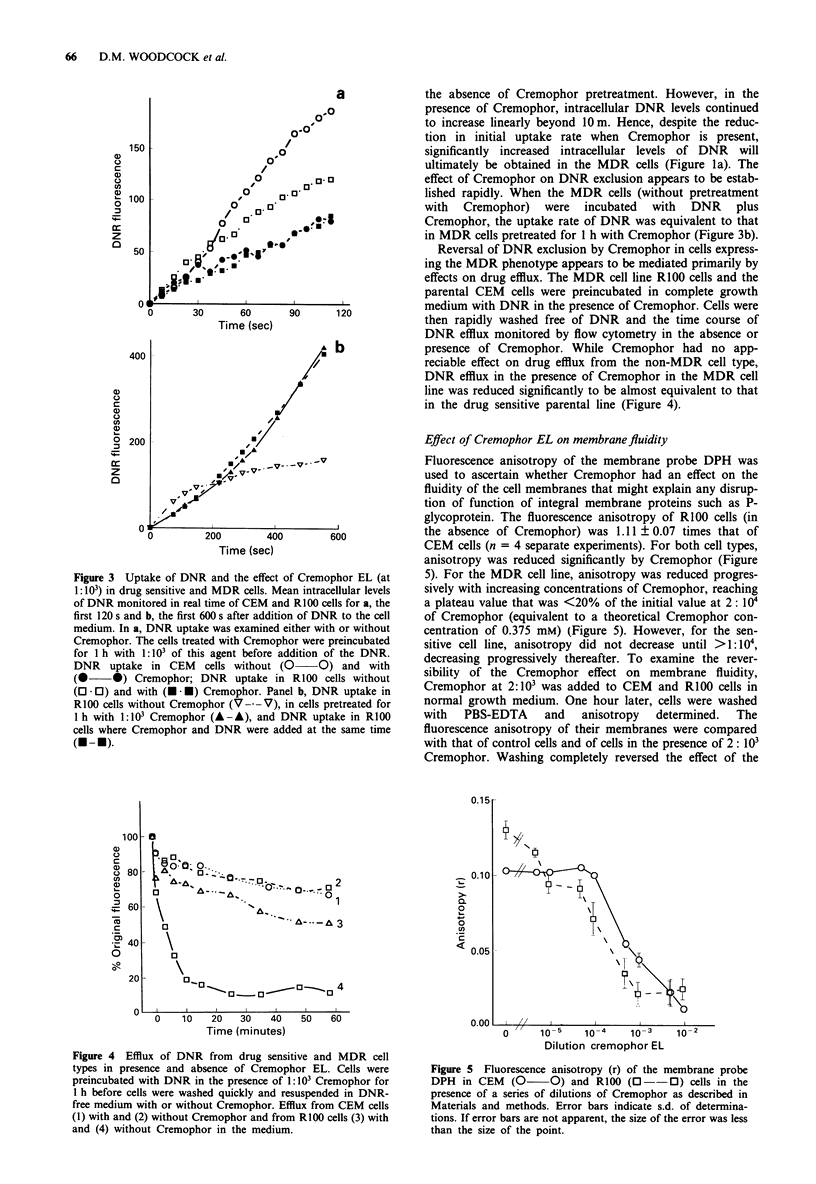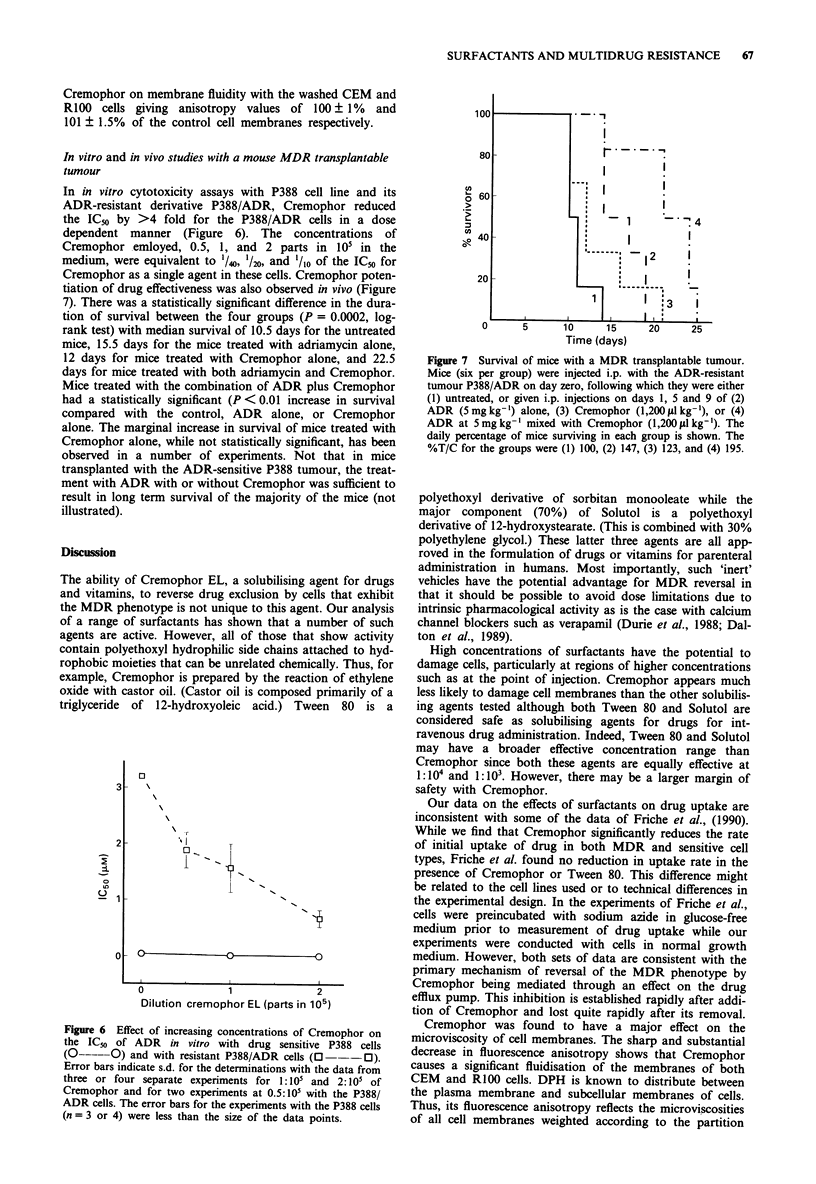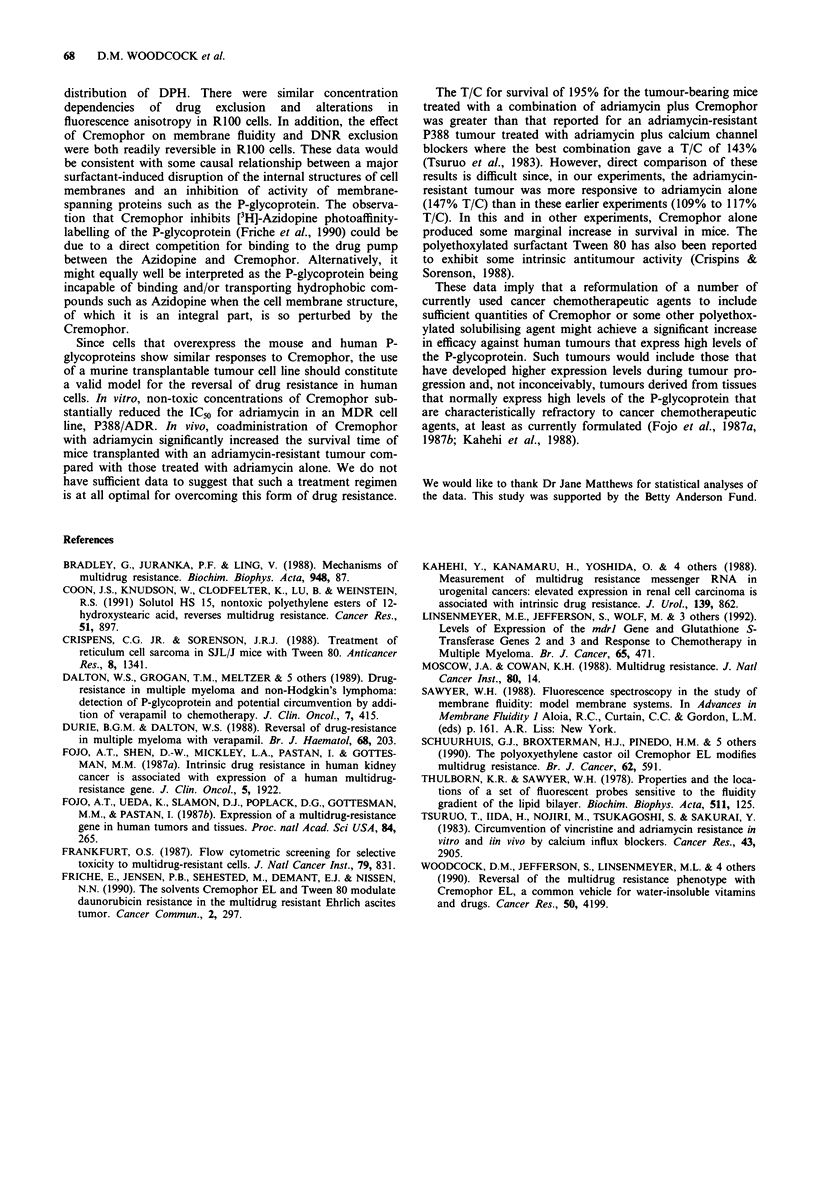# Reversal of multidrug resistance by surfactants.

**DOI:** 10.1038/bjc.1992.217

**Published:** 1992-07

**Authors:** D. M. Woodcock, M. E. Linsenmeyer, G. Chojnowski, A. B. Kriegler, V. Nink, L. K. Webster, W. H. Sawyer

**Affiliations:** Peter MaCallum Cancer Institute, Melbourne, Victoria, Australia.

## Abstract

Cremophor EL, a pharmacologically inactive solubilising agent, has been shown to reverse multidrug resistance (MDR). Using flow cytometric evaluation of equilibrium intracellular levels of daunorubicin (DNR), we found that eight other surface active agents will also reverse MDR. All the active detergents contain polyethoxylated moieties but have no similarities in their hydrophobic components. The properties of three polyethoxylated surfactants that showed the lowest toxicities, Cremophor, Tween 80 and Solutol HS15, were examined in more detail. The concentrations of Tween 80 and Solutol required to reverse DNR exclusion were 10-fold lower than for Cremophor. However while concentrations greater than or equal to 1:10(2) of the former two surfactants resulted in breakdown of cells, even 1:10 of Cremophor did not lyse cells. Studies of the effects of Cremophor on the uptake and efflux of DNR in normal and MDR cell types showed that Cremophor increases intracellular DNR primarily by locking the rapid efflux from the cells. This blockage of drug efflux may be mediated by a substantial alteration in the fluidity of cell membranes induced by Cremophor, as shown by decreased fluorescence anisotropy of a membrane probe. Consistent with these data, coinjection of adriamycin plus Cremophor into mice carrying a multidrug resistant P388 transplantable tumour significantly increased the survival time of the mice compared with adriamycin treatment alone.


Br. J. Cancer (1992), 66, 62–68 © Macmillan Press Ltd., 1992

### Reversal of multidrug resistance by surfactants

[1]D.M. Woodcock[1], M.E. Linsenmeyer[1], G. Chojnowski[1], A.B. Kriegler[1], V. Nink[1], L.K. Webster[2] & W.H. Sawyer[2]

[1]*Peter MaCallum Cancer Institute, Melbourne, Victoria 3000, and* [2]*Department of Biochemistry, University of Melbourne, Parkville, Victoria 3052, Australia.*

**Summary** Cremophor EL, a pharmacologically inactive solubilising agent, has been shown to reverse multidrug resistance (MDR). Using flow cytometric evaluation of equilibrium intracellular levels of daunorubicin (DNR), we found that eight other surface active agents will also reverse MDR. All the active detergents contain polyethoxyl moieties but have no similarities in their hydrophobic components. The properties of three polyethoxylated surfactants that showed the lowest toxicities, Cremophor, Tween 80 and Solutol HS15, were examined in more detail. The concentrations of Tween 80 and Solutol required to reverse DNR exclusion were 10-fold lower than for Cremophor. However while concentrations $\geqslant 1:10^2$ of the former two surfactants resulted in breakdown of cells, even 1:10 of Cremophor did not lyse cells. Studies of the effects of Cremophor on the uptake and efflux of DNR in normal and MDR cell types showed that Cremophor increases intracellular DNR primarily by locking the rapid efflux from the cells. This blockage of drug efflux may be mediated by a substantial alteration in the fluidity of cell membranes induced by Cremophor, as shown by decreased fluorescence anisotropy of a membrane probe. Consistent with these data, coinjection of adriamycin plus Cremophor into mice carrying a multidrug resistant P388 transplantable tumour significantly increased the survival time of the mice compared with adriamycin treatment alone.

Mammalian cells that exhibit the Multidrug Resistance or MDR phenotype produce high levels of a membrane protein (the P-glycoprotein) that acts as a broad spectrum pump that removes from the cell a number of cancer chemotherapeutic agents (reviewed by Bradley *et al.*, 1988 and Moscow *et al.*, 1988). This phenotype has been implicated in intrinsic and acquired drug resistance in a number of human tumours (Fojo *et al.*, 1987*a*, 1987*b*; Kahehi *et al.*, 1988; Linsenmeyer *et al.*, 1992). While a number of agents have been shown to reverse the MDR phenotype, their use in the clinical situation with resistant tumours has been restricted due to dose-limiting toxicities (Dalton *et al.*, 1989: Durie *et al.*, 1988).

Recently, we found that a relatively pharmacologically inert substance, Cremophor EL, will reverse the MDR phenotype in cells in culture at concentrations likely to be readily achievable clinically (Woodcock *et al.*, 1990). Our conclusions have since been confirmed by other studies (Schuurhuis *et al.*, 1990; Friche *et al.*, 1990). Cremophor EL is a polyethylene oxide modified castor oil used as a solubilising agent for drugs and vitamins for both oral and parenteral administration. We have examined a chemically diverse selection of surface active agents to define the structural requirements for MDR reversal by Cremophor El through determining its effects on uptake and efflux of a fluorescent drug eliminated by cells containing an active P-glycoprotein pump. We have also examined the effect of Cremophor EL on the fluidity of cell membranes. Finally, we report the effect of coadministration of Cremophor EL on the survival time of mice bearing an MDR tumour treated with a chemotherapeutic drug affected by the MDR phenotype.

### Materials and methods

### Materials

Cremophor EL and Solutol HS15 were obtained from BASF Fine Chemicals (Melbourne, Australia and Ludwigshafen, Germany, respectively). Detergents #1 to #12 (Table I) were a set supplied by Boehringer Mannheim GmbH, Germany (Cat no. 1124714). The remaining detergents were obtained from Sigma Chemical Co, St. Louis, MO. Chemotherapeutic drugs used in this study were vinblastine (VLB) (Velbe; Lilly), daunorubicin (DNR) (Cerubidin; May & Baker), and adriamycin (ADR)(doxorubicin hydrochloride; Farmitalia Carlo Erba; lyophilised powder, reconstituted in lactose U.S.P.). Mice were obtained from the Animal Resource Centre (Perth, Australia). The P388 and P388/ADR cells were obtained from the National Cancer Institute, USA, and the Cancer Research Laboratory, Auckland Medical School, New Zealand, respectively.

### Cell culture and assay methods

The human leukaemic cell line CCRF-CEM and its MDR-derivative, R100 cells, were maintained in the alpha-modification of Eagle's Minimal Essential Medium (α-MEM) with 10% newborn calf serum (NBCS) (Flow Laboratories, Melbourne, Australia). The R100 cells were cultured in the presence of 100 ng ml$^{-1}$ of VLB for 5 days per month. Estimations of equilibrium intracellular daunorubicin levels were determined by the method of Frankfurt (1987) on an Ortho Diagnostics System 50H Cytofluorograph or Becton Dickinson FACStar Plus Flow Cytometer. Both instruments utilised an argon ion laser operated at 200 mW power excited at 488 nm. Filter sets were 630 nm long pass for the System 50H and 549-601 nm for the FACStar Plus. Cellular integrity in the presence of different concentrations of surfactants was monitored by incubating cells for 1 h in the indicated concentrations of the agent and analysing cell populations by flow cytometry with respect to two physical parameters indicative of cell size and morphology. This allowed gating of intact cells as distinct from cell debris. The actual physical parameters depended on the machine used in the experiment (see caption to Figure 2). Studies of the uptake rates of DNR and the effects of Cremophor used the Chronys software package on the Becton Dickinson instrument. Efflux studies were performed on the Ortho instrument by acquiring data over short defined intervals after the cells had been rapidly washed free of drug.

### *Determination of* in vitro *and* in vivo *toxicity of detergents*

The murine bone marrow-derived cell line, FDC-$P_1$, was grown in α-MEM with 10% foetal calf serum supplemented with 10% of the same medium preconditioned by growth of WEHI-3B cells. The toxicity of various agents was determined by incubating the FDC-$P_1$ cells (1000 ml$^{-1}$) in phos-

Correspondence: D.M Woodcock, Molecular Genetics, Peter MacCallum Cancer Institute, 481 Little Lonsdale Street, Melbourne, Victoria 3000, Australia.
Received 73 October 1991; and in revised form 27 January 1992.

**Table I** Reversal of MDR by various detergents

| | *Detergent*[a] *(diluted 1:10,000)* | *Daunorubicin*[b] *Uptake by R100 Cells (% of CEM)* | $IC_{50}$[c] *(mg ml$^{-1}$)* |
|---|---|---|---|
| | None | 19.9 ± 0.6 | N.D.[d] |
| 1. | n-Octylglucoside | 19.0 ± 3.1 | N.D. |
| 2. | n-Dodecylglucoside | 17.3 ± 0.4 | N.D. |
| 3. | n-Dodecyl-β-D-maltoside | 16.9 ± 8.5 | N.D. |
| 4. | Octanoyl-N-methylglucamide (MEGA-8) | 14.4 ± 0.6 | N.D. |
| 5. | Decanoyl-N-methylglucamide (MEGA-10) | 20.6 ± 0.1 | N.D. |
| 6. | Octylphenolpoly(ethylene-glycolether)$_{10}$ (Triton X-100) | 45.3 ± 5.0 | <0.25 |
| 7. | Octylphenolpoly(ethylene-glycolether)$_7$ (Triton X-114) | 41.4 ± 3.7 | <0.25 |
| 8. | Dodecylpoly(ethylene-glycolether)$_9$ (Thesit) | 57.0 ± 0.3 | <0.25 |
| 9. | Isotridecylpoly(ethylene-glycolether)$_n$ | 35.8 ± 1.8 | <0.25 |
| 10. | 3-[(3-Cholamidopropyl)-dimethylammoniol]-1-propane-sulfonate (CHAPS). | 16.7 ± 0.8 | N.D. |
| 11. | 3-[(3-Cholamidopropyl)-dimethylammonio]-2-propane-sulfonate (CHAPSO) | 16.4 ± 0.4 | N.D. |
| 12. | N-Dodecyl-N,N-dimethyl-3-ammonio-1-propane-sulfonate | 16.1 ± 4.7 | N.D. |
| 13. | Lubrol (Ethylene oxide condensates of fatty alcohols) | 43.5 ± 3.7 | <0.25 |
| 14. | Monolaurylpolyethylene-glycolether-sorbitan (Tween 20) | 38.3 ± 5.4 | 0.25 |
| 15. | Monooleylpolyethyleneglycol-ether-sorbitan (Tween 80) | 50.9 ± 7.0 | 0.31 |
| 16. | Cremophor EL (Polyethoxylated castor oil) | 47.3 ± 0.5 | 5.0 |
| 17. | Polyethylene glycol 4000 | 14.5 ± 0.4 | N.D. |

[a]Diluted in saline (w/v). [b]Each value is the mean ± s.d. of duplicate samples within one experiment. Equilibrium intracellular DNR levels in the MDR cells (R100) in PBS as determined by flow cytometry relative to that observed with drug sensitive CEM cells that was taken as 100%. The data presented here differ from that presented in Figure 1 because serum-free conditions alter the concentration dependency of reversal by surfactants. [c]Dose of agent required to produce a 50% reduction in the cloning efficiency of FDCP-$P_1$ cells following a 2 h incubation as determined by the number of colonies formed after growth for 7 days in agar cultures. [d]N.D. = not determined.

phate buffered saline (PBS) containing the agent to be tested at dilutions of 1:100, 1:200 or 1:400 (w/v) for 2 h at 37°C. The cells were then washed three times with PBS containing 2% NBCS and grown in agar culture 35 mm petri dishes containing 100 cells, 1 ml of 0.33% agar in α-MEM supplemented with 20% NBCS and an optimal dose of recombinant murine granulocyte-macrophage colony-stimulating factor. After incubation for 7 days at 37°C with a gas phase of 7% $O_2$, 10% $CO_2$, 83% $N_2$, the numbers of colonies were counted. The toxicity was expressed as the dose of agent required to reduce the cloning efficiency by 50% ($IC_{50}$).

The *in vivo* toxic effects of various agents were tested in 12 week old male $B_6D_2F_1$ mice. Mice (2) were injected intravenously with the detergent at 25 mg kg$^{-1}$ in saline and observed for immediate shock reactions, presenting as a temporary paralysis. Agents that showed no effect were then tested at successively increasing dosages (33, 50, 100 and 200 mg kg$^{-1}$) until a shock reaction occurred.

### *Mouse P388 tumour* in vitro *and* in vivo

For *in vitro* cytotoxicity experiments, the P388 murine leukaemia and its ADR-resistant subline, P388/ADR, were maintained in RPMI medium with 10% foetal calf serum, 1% glutamine, and 50 μM β-mercaptoethanol. Cells in log phase were diluted to 5 × 10$^4$ ml$^{-1}$ and 2 ml added to wells containing the requisite concentrations of drug and Cremophor with duplicate wells for each combination in each experiment. After 48 h incubation, cells were placed on ice and the cell density in each well determined using a Coulter Counter. For the transplantable tumour model, male mice, aged 12 weeks, were maintained on a standard laboratory diet under a 12 h light/dark cycle and were acclimatised for at least one week prior to use. P388 and P338/ADR cell lines were passaged weekly by intraperitoneal (i.p.) injection of 10$^5$ cells into DBA/2 mice, and were reinitiated from frozen stocks after 20 (P388) or ten (P388/ADR) passages. For *in vivo* assessment of the effectiveness of different drug combinations, groups of six $B_6D_2F_1$ mice were innoculated i.p. with 10$^6$ cells on day 0. Drug treatments were given i.p. on days 1, 5 and 9. Survival was monitored for up to 30 days. Significance levels for the comparisons of survival data were based on the logrank test using 2-tailed tests and not adjusting for multiple comparisons. They were calculated using the BMDP 1988 software. %T/C, the mean survival time of treated mice compared to control mice expressed as a percentage, was also calculated for comparison with other studies.

### *Microviscosity determination*

The fluidity of cell membranes was determined by measuring the fluorescence polarisation of the hydrophobic probe, 1,6-diphenyl-1,3,5-hexatriene (DPH). An aliquot (25 μl) of a stock solution of DPH (2 mM in tetrahydrofuran) was diluted 1:2000 into the suspension buffer (free of BSA and divalent ions) with vigorous mixing. This dispersion was mixed with the cell suspension and a given concentration of Cremophor in PBS, the final cell density being $0.5 \times 10^6 ml^{-1}h$. Probe uptake was for 1 h at room temperature in the dark. Fluorescence polarisation measurements, corrected for the scatter of the excitation beam, were made with a T-format fluorimeter as previously described (Thulborn & Sawyer, 1978). The results are expressed as fluorescence anisotropy, $r = (V-H)/(V+2H)$, where V and H are the intensities of the vertical and horizontal components of the emitted light respectively. No attempt was made to convert the anisotropy values into microviscosities because of the assumptions necessary in that conversion (Sawyer, 1988).

### Results

### *Comparison of detergents*

The activity of the P-glycoprotein pump in cells in culture was assayed by measuring the intracellular level of the fluorescent drug DNR that is excluded by cells actively expressing the MDR phenotype. Reversal of the MDR phenotype causes increased intracellular DNR concentrations that can be readily measured by flow cytometry (Frankfurt, 1987). A range of different detergents was compared with Cremophor for their ability to reverse MDR (Table I). All the detergents with polyethylene oxide hydrophilic moieties like Cremophor (detergents #6-#9 and #13-#15) were able to reverse MDR. Detergents with other types of hydrophilic moieties (detergents #1 to #5 and #10 to #12) did not reverse DNR exclusion at a dilution of 1:10,000. At higher concentrations (1:1000 and 1:2000), these detergents remained ineffective (data not shown) and were in some cases very cytotoxic (detergents #3 and #12). Of those surfactants that showed MDR reversal activity, the concentrations required to cause a 50% reduction in the cloning efficiency of a murine bone marrow-derived cell line, $FDC,P_1$, were $\leqslant 0.31 mg ml^{-1}$, except for Cremophor where the concentration was 16 fold higher ($5 mg ml^{-1}$). *In vivo* administration of the active agents listed in Table I resulted in shock reactions in mice for Tween 20 and Tween 80 at $100 mg kg^{-1}$, for Lubrol at $50 mg kg^{-1}$, for detergent at #6 at $33 mg kg^{-1}$, and for detergents #7, #8, and #9 at $25 mg kg^{-1}$. No adverse effects of the administration of Cremophor were observed even at $200 mg kg^{-1}$. Also, no adverse reactions were observed with another polyethylated surfactant, Solutol HS15, which Coon *et al.* (1991) have shown to be active in overcoming MDR *in vitro*.

The detailed concentration dependencies for reversal of the MDR phenotype by Cremophor and two other polyethox-

**Figure 1** Effect of Cremophor EL, Tween 80, and Solutol HS15 on DNR equilibrium levels in normal and MDR cells. Drug sensitive CEM cells (O——O) and cells of the MDR derivative line R100 (□– – –□) were incubated for 1 h in the presence of the fluorescent drug DNR and the equilibrium intracellular drug level determined by flow cytometry. In **a**, incubations included a range of dilutions of Cremophor. The point 'O' indicates the control (no Cremophor). All points are the average ± the s.d. of triplicate or quadruplicate determinations. If no error bars are apparent, the error was smaller than the size of the symbol. The values for intracellular fluorescence are in arbitrary units. In the final point ('Washed'), cells had been incubated with Cremophor at 1:1000 for 1 h, washed, and incubated in Cremophor-free medium with DNR for an additional 1 h before determination of the intracellular drug levels. In panel **b**, cells were incubated with DNR together with a series of dilutions of Tween 80. Panel **c**, illustrates an equivalent experiment with the detergent Solutol. In panel **d**, the cell lines were the mouse leukaemia P388 (O——O) and an MDR derivative, P388/ADR (□– – –□). In this experiment, the effect on DNR levels is due to overexpression of the mouse rather than the human *mdr1* gene.

ylated solubilising agents, Tween 80 and Solutol HS15 are shown in Figure 1 (Panels a, b and c respectively). (Solutol is primarily polyethoxylated 12-hydroxystearate.) These three surfactants were chosen for more detailed study because they were the least toxic of the surfactants that were active in reversing drug exclusion. The cell types employed were the drug-sensitive human leukaemic cell line CEM and its MDR derivative, R100 cells, that can grow in 100 ng $ml^{-1}$ of VLB and that show a 40 to 50 fold overexpression of the *mdr1* mRNA (Woodcock *et al.*, 1990). While all three agents produced similar maximal intracellular DNR levels, this was achieved at $1:10^4$ with Tween-80 and Solutol but required $1:10^3$ of Cremophor. These experiments involve reversal of the MDR phenotype in a human cell line that has amplified and overexpresses the human *mdr1* gene. This effect of Cremophor on MDR is not cell line or species specific since a similar concentration dependency of MDR reversal was observed with a multidrug resistant mouse cell line, P388/ADR (Figure 1d). Quantitation of *mdr1* mRNA by slot blot analysis indicated a 15 fold higher level of transcription in the P388/ADR cells compared to the P388 parental cells (not illustrated).

Cells recover rapidly from the effect of Cremophor on reversal of DNR exclusion. The drug resistant cells were incubated for 1 h with the optimal dilution of Cremophor (1:1000), washed, and incubated for an additional 1 h in medium containing DNR but no Cremophor. The subsequent intracellular DNR levels were not significantly different from those in control cells that had not been exposed to Cremophor (Figure 1a). Hence by 1 h after removal of the Cremophor, the MDR cells had recovered the ability to exclude drug.

The reduction in DNR levels in cells incubated in higher concentrations of Tween 80 (Figure 1b) was not due primarily to inhibition of uptake but rather to gross cellular damage. When the integrity of cells in the presence of Cremophor and Tween 80 was examined (Figure 2A), it was found that R100 cells remain intact in as much as 1:10 of Cremophor while, with Tween 80, ⅔ of the cells were lysed by 1:100 and all were lysed by 1:10. With these two agents, the CEM cells displayed similar concentration dependency on cell integrity (not illustrated). With the third polyethoxylated solubilising agent, Solutol, R100 cells exhibited similar sensitivity to that found for Tween 80 with ⅔ of the cells lysed by 1 : 100 of this agent (Figure 2B, bottom row). However, CEM cells were found to be more sensitive than the MDR derivative to the lytic effects of Solutol (Figure 2B, top row).

### *Uptake and efflux studies*

The effect of Cremophor on the rate of DNR uptake in drug sensitive and resistant human cells was monitored by continuous assessment of mean intracellular DNR fluorescence over 120 s (Figure 3a) or 600 s (Figure 3b) following addition of DNR to the cells. The initial rate of DNR uptake in the absence of Cremophor was equivalent in both cell lines except that, after 2 to 3 min, the intracellular DNR levels in the MDR cells began to plateau (Figure 3a and 3b). In the presence of Cremophor, initial uptake rate was reduced in both cell types, but total uptake continued to increase in the MDR cell line (Figure 3a). This could be seen more clearly when intracellular DNR levels were monitored over a longer time interval (Figure 3b). Thus, in the R100 cells, a low equilibrium level of intracellular DNR was reached rapidly in

**Figure 2** Effects of Cremophor EL, Tween 80, and Solutol HS15 on cellular integrity. In 2A, R100 cells were incubated with a series of dilutions of Tween 80 (upper panels) or Cremophor (lower panels) in growth medium for 1 h before analysis of cellular integrity by flow cytometry. **a**, Control cells (no detergent), **b**, cells incubated with $1:10^4$ of detergent in the medium, **c**, detergent at $1:10^3$, **d**, detergent at $1:10^2$, and **e**, detergent at 1:10. Intact cells (analysed on an Ortho System 50 by axial light loss *vs* 90° scatter) are present in a defined region of the distribution. Lysed cells disappear from this region while cell debris appears as smaller particles. In **2B**, CEM (upper panels) and R100 cells (lower panels) were incubated for 1 h with a series of dilutions of Solutol. **a**, Control (no detergent), **b**, $1:10^5$ Solutol, **c**, $1:10^4$, **d**, $1:10^3$, and **e**, $1:10^2$. In this experiment, cellular integrity was monitored by examining forward *vs* side scatter on a FACStar Plus flow cytometer.

**Figure 3** Uptake of DNR and the effect of Cremophor EL (at $1:10^3$) in drug sensitive and MDR cells. Mean intracellular levels of DNR monitored in real time of CEM and R100 cells for **a**, the first 120 s and **b**, the first 600 s after addition of DNR to the cell medium. In **a**, DNR uptake was examined either with or without Cremophor. The cells treated with Cremophor were preincubated for 1 h with $1:10^3$ of this agent before addition of the DNR. DNR uptake in CEM cells without (○——○) and with (●——●) Cremophor; DNR uptake in R100 cells without (□·□) and with (■·■) Cremophor. Panel **b**, DNR uptake in R100 cells without Cremophor (▽–·–▽), in cells pretreated for 1 h with $1:10^3$ Cremophor (▲–▲), and DNR uptake in R100 cells where Cremophor and DNR were added at the same time (■–■).

the absence of Cremophor pretreatment. However, in the presence of Cremophor, intracellular DNR levels continued to increase linearly beyond 10 m. Hence, despite the reduction in initial uptake rate when Cremophor is present, significantly increased intracellular levels of DNR will ultimately be obtained in the MDR cells (Figure 1a). The effect of Cremophor on DNR exclusion appears to be established rapidly. When the MDR cells (without pretreatment with Cremophor) were incubated with DNR plus Cremophor, the uptake rate of DNR was equivalent to that in MDR cells pretreated for 1 h with Cremophor (Figure 3b).

Reversal of DNR exclusion by Cremophor in cells expressing the MDR phenotype appears to be mediated primarily by effects on drug efflux. The MDR cell line R100 cells and the parental CEM cells were preincubated in complete growth medium with DNR in the presence of Cremophor. Cells were then rapidly washed free of DNR and the time course of DNR efflux monitored by flow cytometry in the absence or presence of Cremophor. While Cremophor had no appreciable effect on drug efflux from the non-MDR cell type, DNR efflux in the presence of Cremophor in the MDR cell line was reduced significantly to be almost equivalent to that in the drug sensitive parental line (Figure 4).

*Effect of Cremophor EL on membrane fluidity*

Fluorescence anisotropy of the membrane probe DPH was used to ascertain whether Cremophor had an effect on the fluidity of the cell membranes that might explain any disruption of function of integral membrane proteins such as P-glycoprotein. The fluorescence anisotropy of R100 cells (in the absence of Cremophor) was $1.11 \pm 0.07$ times that of CEM cells ($n$ = 4 separate experiments). For both cell types, anisotropy was reduced significantly by Cremophor (Figure 5). For the MDR cell line, anisotropy was reduced progressively with increasing concentrations of Cremophor, reaching a plateau value that was <20% of the initial value at $2:10^4$ of Cremophor (equivalent to a theoretical Cremophor concentration of 0.375 mM) (Figure 5). However, for the sensitive cell line, anisotropy did not decrease until $>1:10^4$, decreasing progressively thereafter. To examine the reversibility of the Cremophor effect on membrane fluidity, Cremophor at $2:10^3$ was added to CEM and R100 cells in normal growth medium. One hour later, cells were washed with PBS-EDTA and anisotropy determined. The fluorescence anisotropy of their membranes were compared with that of control cells and of cells in the presence of $2:10^3$ Cremophor. Washing completely reversed the effect of the

**Figure 4** Efflux of DNR from drug sensitive and MDR cell types in presence and absence of Cremophor EL. Cells were preincubated with DNR in the presence of $1:10^3$ Cremophor for 1 h before cells were washed quickly and resuspended in DNR-free medium with or without Cremophor. Efflux from CEM cells (1) with and (2) without Cremophor and from R100 cells (3) with and (4) without Cremophor in the medium.

**Figure 5** Fluorescence anisotropy (r) of the membrane probe DPH in CEM (○——○) and R100 (□——□) cells in the presence of a series of dilutions of Cremophor as described in Materials and methods. Error bars indicate s.d. of determinations. If error bars are not apparent, the size of the error was less than the size of the point.

Cremophor on membrane fluidity with the washed CEM and R100 cells giving anisotropy values of 100 ± 1% and 101 ± 1.5% of the control cell membranes respectively.

### *In vitro* and *in vivo* studies with a mouse MDR transplantable tumour

In *in vitro* cytotoxicity assays with P388 cell line and its ADR-resistant derivative P388/ADR, Cremophor reduced the $IC_{50}$ by >4 fold for the P388/ADR cells in a dose dependent manner (Figure 6). The concentrations of Cremophor emloyed, 0.5, 1, and 2 parts in $10^5$ in the medium, were equivalent to $^1/_{40}$, $^1/_{20}$, and $^1/_{10}$ of the $IC_{50}$ for Cremophor as a single agent in these cells. Cremophor potentiation of drug effectiveness was also observed *in vivo* (Figure 7). There was a statistically significant difference in the duration of survival between the four groups ($P = 0.0002$, log-rank test) with median survival of 10.5 days for the untreated mice, 15.5 days for the mice treated with adriamycin alone, 12 days for mice treated with Cremophor alone, and 22.5 days for mice treated with both adriamycin and Cremophor. Mice treated with the combination of ADR plus Cremophor had a statistically significant ($P < 0.01$ increase in survival compared with the control, ADR alone, or Cremophor alone. The marginal increase in survival of mice treated with Cremophor alone, while not statistically significant, has been observed in a number of experiments. Not that in mice transplanted with the ADR-sensitive P388 tumour, the treatment with ADR with or without Cremophor was sufficient to result in long term survival of the majority of the mice (not illustrated).

### Discussion

The ability of Cremophor EL, a solubilising agent for drugs and vitamins, to reverse drug exclusion by cells that exhibit the MDR phenotype is not unique to this agent. Our analysis of a range of surfactants has shown that a number of such agents are active. However, all of those that show activity contain polyethoxyl hydrophilic side chains attached to hydrophobic moieties that can be unrelated chemically. Thus, for example, Cremophor is prepared by the reaction of ethylene oxide with castor oil. (Castor oil is composed primarily of a triglyceride of 12-hydroxyoleic acid.) Tween 80 is a

**Figure 6** Effect of increasing concentrations of Cremophor on the $IC_{50}$ of ADR *in vitro* with drug sensitive P388 cells (O——O) and with resistant P388/ADR cells (□——□). Error bars indicate s.d. for the determinations with the data from three or four separate experiments for 1:$10^5$ and 2:$10^5$ of Cremophor and for two experiments at 0.5:$10^5$ with the P388/ADR cells. The error bars for the experiments with the P388 cells ($n = 3$ or 4) were less than the size of the data points.

**Figure 7** Survival of mice with a MDR transplantable tumour. Mice (six per group) were injected i.p. with the ADR-resistant tumour P388/ADR on day zero, following which they were either (1) untreated, or given i.p. injections on days 1, 5 and 9 of (2) ADR (5 mg $kg^{-1}$) alone, (3) Cremophor (1,200 μl $kg^{-1}$), or (4) ADR at 5 mg $kg^{-1}$ mixed with Cremophor (1,200 μl $kg^{-1}$). The daily percentage of mice surviving in each group is shown. The %T/C for the groups were (1) 100, (2) 147, (3) 123, and (4) 195.

polyethoxyl derivative of sorbitan monooleate while the major component (70%) of Solutol is a polyethoxyl derivative of 12-hydroxystearate. (This is combined with 30% polyethylene glycol.) These latter three agents are all approved in the formulation of drugs or vitamins for parenteral administration in humans. Most importantly, such 'inert' vehicles have the potential advantage for MDR reversal in that it should be possible to avoid dose limitations due to intrinsic pharmacological activity as is the case with calcium channel blockers such as verapamil (Durie *et al.*, 1988; Dalton *et al.*, 1989).

High concentrations of surfactants have the potential to damage cells, particularly at regions of higher concentrations such as at the point of injection. Cremophor appears much less likely to damage cell membranes than the other solubilising agents tested although both Tween 80 and Solutol are considered safe as solubilising agents for drugs for intravenous drug administration. Indeed, Tween 80 and Solutol may have a broader effective concentration range than Cremophor since both these agents are equally effective at 1:$10^4$ and 1:$10^3$. However, there may be a larger margin of safety with Cremophor.

Our data on the effects of surfactants on drug uptake are inconsistent with some of the data of Friche *et al.*, (1990). While we find that Cremophor significantly reduces the rate of initial uptake of drug in both MDR and sensitive cell types, Friche *et al.* found no reduction in uptake rate in the presence of Cremophor or Tween 80. This difference might be related to the cell lines used or to technical differences in the experimental design. In the experiments of Friche *et al.*, cells were preincubated with sodium azide in glucose-free medium prior to measurement of drug uptake while our experiments were conducted with cells in normal growth medium. However, both sets of data are consistent with the primary mechanism of reversal of the MDR phenotype by Cremophor being mediated through an effect on the drug efflux pump. This inhibition is established rapidly after addition of Cremophor and lost quite rapidly after its removal.

Cremophor was found to have a major effect on the microviscosity of cell membranes. The sharp and substantial decrease in fluorescence anisotropy shows that Cremophor causes a significant fluidisation of the membranes of both CEM and R100 cells. DPH is known to distribute between the plasma membrane and subcellular membranes of cells. Thus, its fluorescence anisotropy reflects the microviscosities of all cell membranes weighted according to the partition distribution of DPH. There were similar concentration dependencies of drug exclusion and alterations in fluorescence anisotropy in R100 cells. In addition, the effect of Cremophor on membrane fluidity and DNR exclusion were both readily reversible in R100 cells. These data would be consistent with some causal relationship between a major surfactant-induced disruption of the internal structures of cell membranes and an inhibition of activity of membrane-spanning proteins such as the P-glycoprotein. The observation that Cremophor inhibits [$^3$H]-Azidopine photoaffinity-labelling of the P-glycoprotein (Friche *et al.*, 1990) could be due to a direct competition for binding to the drug pump between the Azidopine and Cremophor. Alternatively, it might equally well be interpreted as the P-glycoprotein being incapable of binding and/or transporting hydrophobic compounds such as Azidopine when the cell membrane structure, of which it is an integral part, is so perturbed by the Cremophor.

Since cells that overexpress the mouse and human P-glycoproteins show similar responses to Cremophor, the use of a murine transplantable tumour cell line should constitute a valid model for the reversal of drug resistance in human cells. *In vitro*, non-toxic concentrations of Cremophor substantially reduced the $IC_{50}$ for adriamycin in an MDR cell line, P388/ADR. *In vivo*, coadministration of Cremophor with adriamycin significantly increased the survival time of mice transplanted with an adriamycin-resistant tumour compared with those treated with adriamycin alone. We do not have sufficient data to suggest that such a treatment regimen is at all optimal for overcoming this form of drug resistance.

The T/C for survival of 195% for the tumour-bearing mice treated with a combination of adriamycin plus Cremophor was greater than that reported for an adriamycin-resistant P388 tumour treated with adriamycin plus calcium channel blockers where the best combination gave a T/C of 143% (Tsuruo *et al.*, 1983). However, direct comparison of these results is difficult since, in our experiments, the adriamycin-resistant tumour was more responsive to adriamycin alone (147% T/C) than in these earlier experiments (109% to 117% T/C). In this and in other experiments, Cremophor alone produced some marginal increase in survival in mice. The polyethoxylated surfactant Tween 80 has also been reported to exhibit some intrinsic antitumour activity (Crispins & Sorenson, 1988).

These data imply that a reformulation of a number of currently used cancer chemotherapeutic agents to include sufficient quantities of Cremophor or some other polyethoxylated solubilising agent might achieve a significant increase in efficacy against human tumours that express high levels of the P-glycoprotein. Such tumours would include those that have developed higher expression levels during tumour progression and, not inconceivably, tumours derived from tissues that normally express high levels of the P-glycoprotein that are characteristically refractory to cancer chemotherapeutic agents, at least as currently formulated (Fojo *et al.*, 1987*a*, 1987*b*; Kahehi *et al.*, 1988).

We would like to thank Dr Jane Matthews for statistical analyses of the data. This study was supported by the Betty Anderson Fund.

### References

BRADLEY, G., JURANKA, P.F. & LING, V. (1988). Mechanisms of multidrug resistance. *Biochim. Biophys. Acta*, **948**, 87.

COON, J.S., KNUDSON, W., CLODFELTER, K., LU, B. & WEINSTEIN, R.S. (1991) Solutol HS 15, nontoxic polyethylene esters of 12-hydroxystearic acid, reverses multidrug resistance. *Cancer Res.*, **51**, 897.

CRISPENS, C.G. JR. & SORENSON, J.R.J. (1988). Treatment of reticulum cell sarcoma in SJL/J mice with Tween 80. *Anticancer Res.*, **8**, 1341.

DALTON, W.S., GROGAN, T.M., MELTZER & 5 others (1989). Drug-resistance in multiple myeloma and non-Hodgkin's lymphoma: detection of P-glycoprotein and potential circumvention by addition of verapamil to chemotherapy. *J. Clin. Oncol.*, **7**, 415.

DURIE, B.G.M. & DALTON, W.S. (1988). Reversal of drug-resistance in multiple myeloma with verapamil. *Br. J. Haematol*, **68**, 203.

FOJO, A.T., SHEN, D.-W., MICKLEY, L.A., PASTAN, I. & GOTTESMAN, M.M. (1987*a*). Intrinsic drug resistance in human kidney cancer is associated with expression of a human multidrug-resistance gene. *J. Clin. Oncol.*, **5**, 1922.

FOJO, A.T., UEDA, K., SLAMON, D.J., POPLACK, D.G., GOTTESMAN, M.M., & PASTAN, I. (1987*b*). Expression of a multidrug-resistance gene in human tumors and tissues. *Proc. natl Acad. Sci USA*, **84**, 265.

FRANKFURT, O.S. (1987). Flow cytometric screening for selective toxicity to multidrug-resistant cells. *J. Natl Cancer Inst.*, **79**, 831.

FRICHE, E., JENSEN, P.B., SEHESTED, M., DEMANT, E.J. & NISSEN, N.N. (1990). The solvents Cremophor EL and Tween 80 modulate daunorubicin resistance in the multidrug resistant Ehrlich ascites tumor. *Cancer Commun.*, **2**, 297.

KAHEHI, Y., KANAMARU, H., YOSHIDA, O. & 4 others (1988). Measurement of multidrug resistance messenger RNA in urogenital cancers: elevated expression in renal cell carcinoma is associated with intrinsic drug resistance. *J. Urol.*, **139**, 862.

LINSENMEYER, M.E., JEFFERSON, S., WOLF, M. & 3 others (1992). Levels of Expression of the *mdr1* Gene and Glutathione *S*-Transferase Genes 2 and 3 and Response to Chemotherapy in Multiple Myeloma. *Br. J. Cancer*, **65**, 471.

MOSCOW, J.A. & COWAN, K.H. (1988). Multidrug resistance. *J. Natl Cancer Inst.*, **80**, 14.

SAWYER, W.H. (1988). Fluorescence spectroscopy in the study of membrane fluidity: model membrane systems. In *Advances in Membrane Fluidity 1* Aloia, R.C., Curtain, C.C. & Gordon, L.M. (eds) p. 161. A.R. Liss: New York.

SCHUURHUIS, G.J., BROXTERMAN, H.J., PINEDO, H.M. & 5 others (1990). The polyoxyethylene castor oil Cremophor EL modifies multidrug resistance. *Br. J. Cancer*, **62**, 591.

THULBORN, K.R. & SAWYER, W.H. (1978). Properties and the locations of a set of fluorescent probes sensitive to the fluidity gradient of the lipid bilayer. *Biochim. Biophys. Acta*, **511**, 125.

TSURUO, T., IIDA, H., NOJIRI, M., TSUKAGOSHI, S. & SAKURAI, Y. (1983). Circumvention of vincristine and adriamycin resistance *in vitro* and *iin vivo* by calcium influx blockers. *Cancer Res.*, **43**, 2905.

WOODCOCK, D.M., JEFFERSON, S., LINSENMEYER, M.L. & 4 others (1990). Reversal of the multidrug resistance phenotype with Cremophor EL, a common vehicle for water-insoluble vitamins and drugs. *Cancer Res.*, **50**, 4199.